# Composite Attention Residual U-Net for Rib Fracture Detection

**DOI:** 10.3390/e25030466

**Published:** 2023-03-07

**Authors:** Xiaoming Wang, Yongxiong Wang

**Affiliations:** Department of Automation, School of Optical-Electrical and Computer Engineering, University of Shanghai for Science and Technology, 516 Jun Gong Road, Yangpu District, Shanghai 200093, China

**Keywords:** U-net, rib fractures, CT, deep learning

## Abstract

Computed tomography (CT) images play a vital role in diagnosing rib fractures and determining the severity of chest trauma. However, quickly and accurately identifying rib fractures in a large number of CT images is an arduous task for radiologists. We propose a U-net-based detection method designed to extract rib fracture features at the pixel level to find rib fractures rapidly and precisely. Two modules are applied to the segmentation network—a combined attention module (CAM) and a hybrid dense dilated convolution module (HDDC). The features of the same layer of the encoder and the decoder are fused through CAM, strengthening the local features of the subtle fracture area and increasing the edge features. HDDC is used between the encoder and decoder to obtain sufficient semantic information. Experiments show that on the public dataset, the model test brings the effects of Recall (81.71%), F1 (81.86%), and Dice (53.28%). Experienced radiologists reach lower false positives for each scan, whereas they have underperforming neural network models in terms of detection sensitivities with a long time diagnosis. With the aid of our model, radiologists can achieve higher detection sensitivities than computer-only or human-only diagnosis.

## 1. Introduction

At present, artificial intelligence technology has developed rapidly in medical image analysis. Deep learning [1] has achieved significant success in classification [2], detection [3,4,5], and segmentation [6,7,8] tasks for 2D and 3D medical images. More and more researchers have started to explore the applications of machine learning methods to medical images and have made apparent progress, such as brain tumor detection [9,10] and lung nodule detection [4]. The segmentation of large organs, such as liver segmentation [6,7], atrial segmentation [11,12], etc., has reached high accuracy.

Rib fractures are a common disease in orthopedics and traumatology, and CT examination is one of the most effective methods for the clinical diagnosis of rib fractures. With the popularity of CT equipment, the burden on orthopedic surgeons to interpret images has increased. Because many rib fractures only have unobservable cracks or differences, the missed diagnosis [13] caused by artificial diagnosis is usually inevitable.

The introduction of machine learning methods for rib detection can effectively reduce the missed diagnosis rate because of doctors’ clinical experience, detection skills, and mental state. Additionally, rib fracture diagnosis is often employed to assess the level of accident injury. Computer-aided diagnosis is expected to improve the accuracy and speed of detection and improve the doctor–patient relationship. Therefore, artificial intelligence for the automatic positioning of rib fractures has vital practical significance.

**Figure 1:**
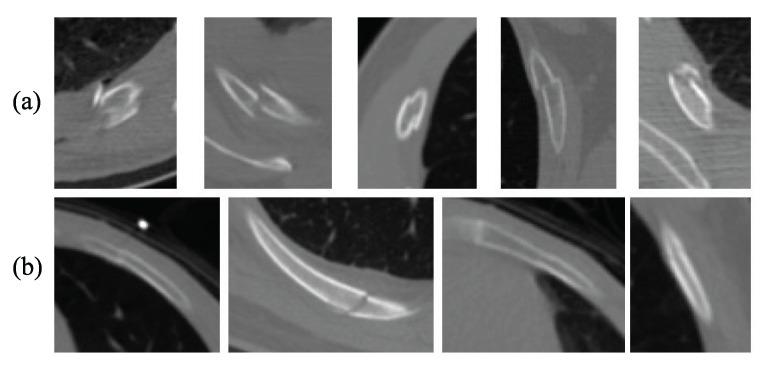
Examples of several common fractures. (**a**) Complete fractures, (**b**) occult fractures.

Some methods have been published for detecting rib fractures in recent years. Gunz et al. [5] unfold the ribs, reconstruct the rib images, and correctly detect the rib fractures using object models. Zhou et al. [14] detect and classify rib fractures using Faster R-CNN two-stage target detection model. Although the two stages improve accuracy, the speed is relatively slow, and it is difficult to achieve the real-time detection effect. Simultaneously, the rib occupies a small area in the axial CT image, and many fracture lines are blurred. As shown in Figure 1, the complete fracture features are apparent, while most of the occult fractures have subtle features that are easily overlooked. Therefore, pixel-level detection is more applicable.

U-net [15] is a classic medical image segmentation model that uses an asymmetric encoded-decoded structure. It skips a connection in the same stage in which multi-scale prediction and deep supervision are performed. U-net is optimal to accelerate the convergence of the neural network and obtain smoother convolution kernels. However, segmentation tasks with small-areas and significant data imbalances have always been a difficult point in deep learning, and this is a problem for U-net as well.

In U-net, low-level features from the bottom layers have rich detail and local information, such as point, line, or edge, but contain complex background information simultaneously. In contrast, high-level features preserve more global features, while low-level features preserve more local ones. We propose a combined attention module (CAM) instead of a direct connection between high-level and low-level features according to the above characteristics. High-level and low-level features condense valuable information through the channel attention mechanism to intensify local features. CAM is beneficial in increasing the microfracture features’ weight and reducing the background information interference.

In addition, dilated convolution is employed to expand the field of convolutional kernels in many image segmentation tasks [16,17]. Wang et al. [18] use a different dilation rate for each layer to solve the problem. Enlightened by the above discussion and the Inception structure [19], we design a HDDC module to enlarge the field of convolutional kernels. Multi-scale dilated convolution operation is performed using a mixed cascade mode to capture deeper and wider semantic features.

Furthermore, rib fractures are often accompanied by changes in the morphology of the surrounding ribs, such as pneumothorax and pleural effusion. The tissue morphology around rib fractures becomes an indirect clue for the network to identify fractures. Therefore, the effect of fracture detection and training based on samples with surrounding tissues is visibly better than that of only rib fractures.

Our contributions are summarized as follows:

1. We design a CAM module integrated with the channel attention mechanism according to the characteristics of high and low-level features for tiny features;
2. Inspired by Inception [19] and hybrid dilated convolution [18], we propose a hybrid dense dilated convolution (HDDC), which is used to mine semantic features and improve the interpretability of the model;
3. We propose a modified U-net network with CAM and HDDC for rib fracture recognition. Our approach outperforms classical semantic segmentation models in each quantitative indicator (F1, precision, Recall, and Dice).

The rest of the paper is organized as follows. Section 2 introduces the related works. Section 3 details the proposed method. Section 4 presents the experimental results and comparison with other networks. In Section 5, we draw some conclusions and offer future research directions.

## 2. Related Work

### 2.1. U-Net Network

The U-net network with the encoder-decoder structure is entirely symmetrical. The up-sampling and down-sampling stages have the same number of layers connected by the skip connection. The skip connection allows the features extracted by the down-sampling layer to be directly concatenated to the up-sampling layer. This unique structure shows a decisive advantage in medical image segmentation, and when processing biomedical datasets with a small amount of data, a better segmentation effect is obtained.

Because of the excellent performance of the U-net network, it has attracted widespread attention in the field of medical image segmentation. Many researchers have optimized this basis and derived many branch networks [20,21,22,23,24]. H-DenseUNet [7] is a novel end-to-end network, including a 2D DenseUNet for extracting intra-slice features and a 3D DenseUNet for aggregating volumetric contexts for liver tumor segmentation. Unet++ [25] is a flexible feature fusion network whose skip connection is redesigned in the decoder sub-network to aggregate features of different semantic scales. Isensee et al. proposed nnU-Net [26], an adaptive framework based on 2D and 3D U-net. The author believes that model performance and generalization are more critical than network design details.

Since the rise of the U-net network, many researchers have improved the U-net to detect rib fractures. Jin et al. [27] designed a novel model improved by 3D U-net, FracNet, which adopted a sampling strategy during training and achieved a high sensitivity. Zhang et al. [28] proposed a rib fracture recognition model, which consists of a nnU-Net [26] as the region segmentation model and a Densenet [29] as the classification model. The two-stage recognition model effectively reduced the FP (false positive) and FN (false negative) rates of rib fracture detection. The above works provide us with referable solutions for detecting rib fractures. However, most of them are carried out on a 3D basis, requiring a high-performance hardware environment and not meeting real-time requirements. For the convenience of training and application, we research a 2D network. We integrate the attention mechanism and hybrid dense dilated convolution into the U-net network with a residual structure to detect rib fractures more accurately.

### 2.2. Inception Modules

Inception modules are layers that perform multiple convolutions of different sizes and pooling operations in parallel. The outputs of these parallel operations are then concatenated and fed into the next layer. The idea behind this design is to capture features of different scales and complexity levels in a single layer, which can help improve the model’s ability to recognize objects of different sizes and shapes in images.

The original Inception model [30] has undergone several iterations since its introduction, with each version adding improvements and optimizations. These later versions include Inception V2 [31], V3 [32], V4 [19], and Inception-ResNet [19], which incorporate additional techniques such as batch normalization, factorized convolutions, and residual connections to improve the performance.

### 2.3. Attention Mechanism

Attention mechanisms have extensive applications in computer vision tasks [33,34,35,36,37,38]. Hu et al. [33] first proposed channel attention, which adaptively recalibrates the weight of each channel. Wang et al. [39] proposed the residual attention network (RAN) by combining a spatial attention mechanism with residual connections.

Some approaches combine spatial and channel attention, allowing the network to focus selectively on both spatial locations and features. CBAM [40] stacks channel attention and spatial attention in series to enhance informative channels and important regions. Zhang et al. [41] leverage self-attention mechanisms for channel and spatial attention to explore pairwise interaction. Roy et al. [42] propose spatial and channel SE blocks (scSE), which are used to provide spatial attention weights to focus on important regions.

These attention mechanisms can be incorporated into various CNN architectures and have been shown to improve the performance on various computer vision tasks.

## 3. Method

### 3.1. The Proposed Model

**Figure 2:**
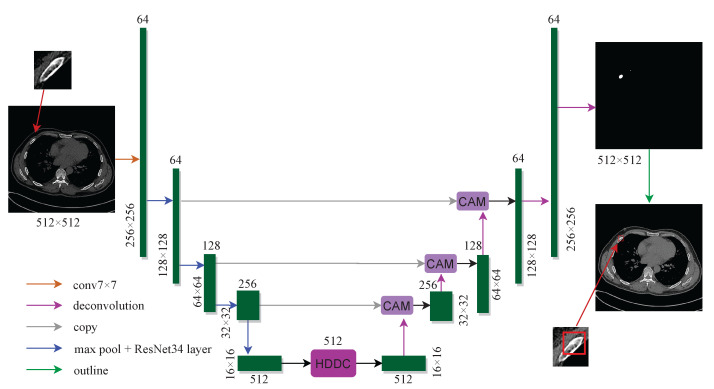
The architecture of the composite attention residual U-net network.

The proposed composite attention residual U-net structure is shown in Figure 2. The network includes two parts: encoding and decoding. The encoding part on the left is responsible for feature extraction. As the network layer deepens, the network channels increase, and the feature map gradually becomes smaller. The function of the decoding part on the right is to restore the features. The corresponding coding layer information is added to the network during decoding to avoid information loss.

In our proposed method, each encoding module in the U-net network is replaced with the corresponding residual module [43] in ResNet34. In contrast to the original module, a direct mapping part is added to the residual module, effectively avoiding gradient explosion and disappearance problems. Besides, we introduce a hybrid dense dilated convolution module after coding and fuse an attention mechanism model in the decoding stage. Finally, the detection bounding box is obtained according to the segmentation results, and the fracture location is marked.

### 3.2. Hybrid Dense Dilated Convolution Module

Dilated convolution injects gaps into the standard convolution map to expand the reception field. In the dilated convolution, the dilation rate indicates the degree of expansion of the convolution kernel (the standard convolution dilation rate is 1).

Unfortunately, the dilated convolution kernel is not continuous. Using the convolution kernel with the same dilated ratio is superimposed multiple times. Some pixels are ignored. In addition, when only a convolution kernel with a large dilation rate is utilized, it will do more harm than good for small objects.

**Figure 3:**
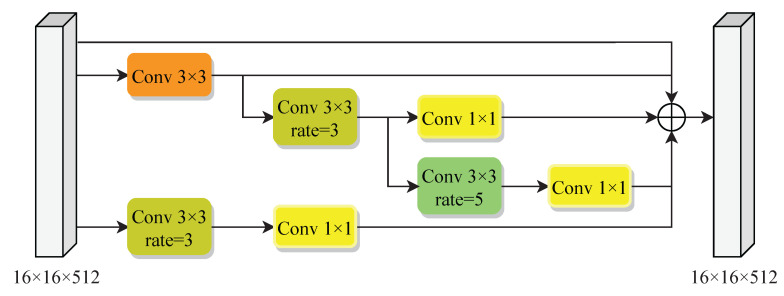
The architecture of the hybrid dilated dense convolution module. It cascades standard and dilated convolution to extract feature information from different scales. Here, all channels are 512, and the rate represents the dilation rate.

Inception [19] is a classical architecture in deep learning. Inception adopts different receptive fields to widen the structure of a network. Inspired by the Inception and hybrid dilated convolution [18], we propose a hybrid dense dilated convolution (HDDC). HDDC, which combines Inception and dilated convolution, inherits the advantages of both approaches. Convolutions of different sizes capture various receptive fields, and features of different scales are merged through the final stitching. For easy alignment, the convolution kernel employs 1 × 1 and 3 × 3. Because the dilation rate increases, the dilated convolution kernel is much larger than the original 3 × 3 convolution kernel, so the 5 × 5 is not employed in HDDC. The detail of HDDC is shown in Figure 3. The final output feature map of the encoding part is processed through several convolutions. These outputs are adjusted to be consistent by 1 × 1 convolution and are then superimposed as the input of the decoding part. HDDC completely captures the object information and effectively reduces the loss of pixel information while expanding the convolution’s receptive field. Meanwhile, more semantic representations are extracted, and then the feature extraction efficiency is improved.

### 3.3. Combined Attention Module

**Figure 4:**
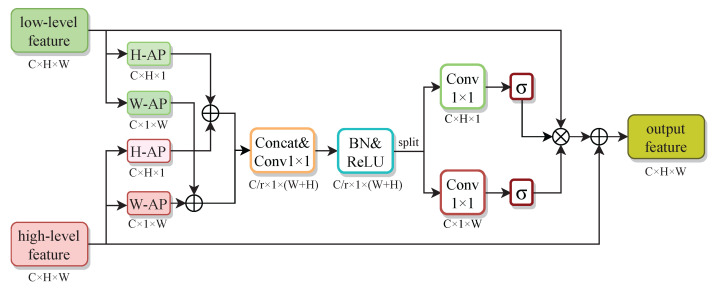
The architecture of the combined attention module (CAM). Attention information from high-level and low-level features is extracted to strengthen the parts that need attention in high-level features. H-AP and W-AP refer to the global average pooling along horizontal and vertical directions.

High-level feature maps contain rich semantic information, while low-level feature maps contain more detailed information. The decoder recovers detailed information through deconvolution upsampling. However, upsampling will cause blurred edges and a loss of detail. Directly connecting low-level and high-level features such as residual networks will bring much background information, which may interfere with the segmentation of the target object. This paper utilizes coordinate attention [44] to integrate high-level and low-level features instead of direct concatenation. The subtle features are strengthened, and the noise interference in the low-level features is reduced. The combined attention module is shown in Figure 4. First, we encode each channel of high-level and low-level features along two directions. The pooling kernels are (*H*, 1) and (1, *W*). These output features are formulated as follows:

(1) $$z_{t}^{h}\left(h\right)=\frac{1}{W}\sum_{0\leq i<W}x_{t}\left(h,i\right)$$

(2) $$z_{l}^{h}\left(h\right)=\frac{1}{W}\sum_{0\leq i<W}x_{l}\left(h,i\right)$$

(3) $$z_{t}^{w}\left(w\right)=\frac{1}{H}\sum_{0\leq j<H}x_{t}\left(j,w\right)$$

(4) $$z_{l}^{w}\left(w\right)=\frac{1}{H}\sum_{0\leq j<H}x_{l}\left(j,w\right)$$

where $x_{t}$ and $x_{l}$ refer to high-level and low-level features, respectively.

The above four operations differ from direct squeeze [33], which captures features along two coordinate directions. By combining the two transformations, long-range spatial dependencies and positional information are preserved along two directions. The concatenation is done following the two levels’ superposition of the two directions. Then, 1 × 1 convolutional function $F_{1\times 1}$ and non-linear activation function δ are executed. The former can be written as

(5) $$y=\delta \left(F_{1\times 1}\left(\mathrm{concat}\left[z_{t}^{h}+z_{l}^{h},z_{t}^{w}+z_{l}^{w}\right]\right)\right)$$

here, $y\in R^{C/r\times (H+W)}$ represents the feature map in a horizontal and vertical orientation as in the coordinate attention block. *r* is the channel compression ratio. Next, the features are split into two direction tensors $y^{w}$ and $y^{h}$.

Two 1 × 1 convolutional functions $F_{1\times 1}^{w}$ and $F_{1\times 1}^{h}$ are applied to get $f^{w}$ and $f^{h}$ with the number of input channels C. The processes can be shown as follows:

(6) $$f^{w}=\sigma \left(F_{1\times 1}^{w}\left(y^{w}\right)\right)$$

(7) $$f^{h}=\sigma \left(F_{1\times 1}^{h}\left(y^{h}\right)\right)$$

here, σ is the sigmoid function.

Finally, attention weights for two directions are enhanced on the low-level features maps and then added to the high-level features maps. The calculation process can be expressed as follow:

(8) $$x_{o}=x_{l}\left(i,j\right)\times f^{h}\left(i\right)\times f^{w}\left(j\right)+x_{t}$$

where $x_{o}$ is the output feature map.

### 3.4. Loss Function

Cross entropy is defined as measuring the difference between two probability distributions for a given random variable or set of events. It is widely used for classification tasks. Since segmentation is pixel-level classification, cross-entropy can also be utilized in segment tasks. Cross entropy loss is defined in Equation (9)

(9) $$L_{CE}=-\frac{1}{w\times h}\sum_{0\leq i<w}\sum_{0\leq j<h}y_{ij}\log\left({\tilde{y}}_{ij}\right)$$

where *w*, *h* denote the width and the height of the input picture. $y_{ij}$ and ${\tilde{y}}_{ij}$ represent the ground truth and the prediction of a pixel, respectively.

The cross-entropy loss function separately evaluates the class prediction of each pixel vector and then averages all pixels from Equation (9), so the pixels in the image are learned equally. The fracture area occupies a small part of the picture in the rib fracture segmentation task. That means the number of negative samples is much greater than the number of positive samples. The components of negative samples in the loss function will dominate, and only the cross-entropy loss makes the model heavily biased towards the background.

Dice coefficient [45], defined as Equation (10), is suitable for highly unbalanced samples, but simple dice loss will adversely affect backpropagation and make training unstable. To effectively use the cross-entropy loss function and the Dice loss function, we combine these two losses as Equation (11).

(10) $$Dice=\frac{2\times \sum_{i=1}^{w}\sum_{j=1}^{h}y_{ij}{\tilde{y}}_{ij}}{\sum_{i=1}^{w}\sum_{j=1}^{h}y_{ij}+\sum_{i=1}^{w}\sum_{j=1}^{h}{\tilde{y}}_{ij}}$$

(11) $$L=\left(1-\theta \right)L_{CE}-\theta \log\left(Dice\right)$$

here, θ is an introduced hyperparameter that can balance Dice loss and cross-entropy loss.

When the prediction deviates far from the ground truth, Dice will be tiny, and the loss will increase to penalize this poor prediction eventually. This method can also improve the sensitivity of loss. This compound loss combines cross-entropy and Dice to maximize strengths and avoid weaknesses. Compared with any loss alone, it has a more remarkable improvement.

## 4. Experiments

### 4.1. Experimental Setup

#### 4.1.1. Datasets

The rib fracture radiography images are from MICCAI 2020 RibFrac Challenge (Rib Fracture Detection and Classification) [27]. The image dataset includes 500 cases of chest-abdomen CT scans. The image-sufficient artificial annotation process participated in the annotation process to ensure higher annotation quality. We divide 420 as a training dataset, and the remaining 80 cases are test sets used for verification. First, the 2D images are extracted from the nii format CT images. For clarity and retaining the tissue voxels around some ribs, the CT image window width is set to 1000, and the window level is set to 600. Images are removed if the total pixel value of the annotated image is less than 100. Therefore, our training dataset has 38,330 2D images (to train the deep learning network), and our test dataset has 5005 2D images (to evaluate the network performance).

The CT detector irradiated the human measured X-ray attenuation coefficient to get the CT value. It is a quantitative density concept used to describe the value density in the CT image, and the unit is HU (Hounsfield Unit). The general practice is to position the water CT value of 0HU, the cortical bone CT value of +1000 Hu, the air CT value of −1000 Hu, and the other tissue between −1000 Hu +1000 Hu. CT images are expressed in different gray levels, reflecting the degree of absorption of X-rays by organs and tissues. The window width, which affects the contrast and sharpness of the image, refers to the range of CT values displayed in the CT image. The window level is the center position of the CT value in the CT image. Suitable window width and window level can reflect the anatomical content and lesion image performance. Here, we set the window width to 1000, and the window level is set to 600.

#### 4.1.2. Experimental Details

These experiments are conducted on the workstation with two INTEL XEON E5-2678 CPUs and two GeForce RTX 2080S GPUs. The deep learning model is trained on the Pytorch framework. The training details are as follows: (1) training with 25 epochs; (2) optimizer that uses stochastic gradient descent (SGD) with 0.0005 weigh decay and 0.9 momentum parameter; (3) batch size, which is set to 16.

#### 4.1.3. Evaluation Metrics

We adopt Precision, Recall, and F1 as the metrics to evaluate our method. When comparing the effect with other networks, we add Dice, as formulated in Equation (10) for evaluation, which is the most popular metric in medical image segmentation. The metrics mentioned above are defined as follows:

(12) $$Precision=\frac{TP}{TP+FP}$$

(13) $$Recall=\frac{TP}{TP+FN}$$

(14) $$F1=\frac{2\times Precision\times Recall}{Precision+Recall}$$

where TP and FN denote the numbers of fractures that are detected correctly or not, respectively. FP represents the number of healthy images that are detected as fractures.

### 4.2. Main Results

#### 4.2.1. Parameter Sensitivity

Our model introduces a new hyper-parameter θ to balance cross-entropy loss and Dice loss. In our experiment, θ is a fixed value, ranging from 0 to 1. When θ is 0, the loss function equals cross-entropy loss. As θ increases, the loss function becomes more and more biased toward Dice loss. When θ is 1, the loss function is entirely equal to log(Dice). Table 1 shows that when θ is 0.2, the model’s performance is the best, and when θ is 0.1, there is a significant fluctuation in the training process, and the training is extremely unstable. When θ ranges from 0.4 to 1.0, fluctuations in the results indicate that the effect of cross-entropy loss is negligible.

#### 4.2.2. Ablation Studies

We evaluate the effect of two modules in the rib fracture dataset in Table 2. (1) HDDC: hybrid dense dilated convolution with multi-scale dilated convolution. (2) CAM: we combine high-level and low-level features in the decoding stage.

Experimental results are shown in Table 2. Unet-34 represents U-net with ResNet34. The context information in the low-level features is integrated into the high-level features by CAM, which helps eliminate some irrelevant information and get strong feature representations (Recall: +5.27%; F1:+2.28%). HDDC improves the performance by 2.85% (Recall) and 2.59% (F1), which shows that the network benefits from multi-scale dilated convolution. The low dilation rate focuses on short-distance information, and the large dilation rate focuses on long-distance details to obtain more features while expanding the receptive field. HDDC enhances the ability to fetch remote information and enables the network to capture more semantic information. We combine the high-level and low-level features to represent multi-scale rib fractures, achieving 81.71% (Recall) and 81.86% (F1).

#### 4.2.3. Comparison with Other Networks

To verify the effectiveness of the network in this paper, we conduct some comparative studies with other state-of-the-art segmentation networks. Considering the fairness of the experiments, the experiments of Unet-34, CE-net, Unet++, and RAUNet adopt the same optimization algorithm, loss function, and initial experimental parameters as the model in this paper. The comparison results are shown in Table 3.

As the basic model, the performance of Unet-34 is the worst. Unet++, which is more complex and has more learnable parameters, performs slightly better than CE-net and RAUNet. Our model only makes local improvements based on Unet-34 without increasing the computational burden too much, and it significantly improves the model performance. In experiments, the Dice similarity coefficient of our algorithm is 53.28%, which is 0.37% higher than that of Unet++. Our model results are the best in terms of Recall, Precision, and F1. It can be concluded that the rib fracture identification of our network is better than other segmentation networks. The significant performance improvement shows that HDDC and CAM have played a vital role.

**Figure 5:**
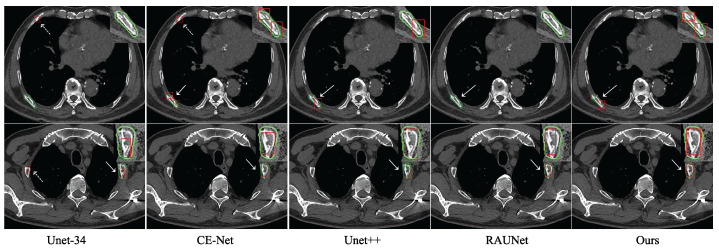
Examples of the detection results of several methods on the validation area in the chest CT images dataset. The green and red boxes denote the contours of the ground truth and detection results, respectively.

For the intuitive comparison, some of the recognition effects of these networks are visualized in Figure 5. Here, the green curve denotes the contour of the ground truth, and the red box marks the location of the rib fracture.

In Figure 5, the Unet-34 network has significantly more missed and false detections than the others. It is clear that the labeling boxes with our method fit more with the ground truth and more completely capture the fracture area. The observation shows the effectiveness of our learning method, i.e., HDDC and CAM. However, some fracture areas in the figure are identified as two areas. This situation shows that identifying fractures by segmentation focuses more on the pixel level. Such parts can be merged through image post-processing as needed.

## 5. Discussion

This paper proposes a deep learning model-based 2D U-net network to detect and segment rib fractures from CT. Through CAM, features from the encoder and the decoder are combined, allowing for the detection of subtle features of occult fractures. HDDC is used between the encoder and decoder to expand the convolutional receptive field through multi-scale cascaded dilated convolution kernels, extract rich semantic features, and improve fracture recognition accuracy.

In detection, our model achieved recall (81.71%) and FPs (25.41), outperforming the average of human experts (about 77.5%, 1.13) [27]. Besides, our network performed 53.28% in Dice, which was acceptable on 2D rib fracture segmentation.

Prior to our study, there were two deep learning-based rib fracture detection models that performed well. Zhou et al. [14] presented a rib fractures detection and classification model based on Faster R-CNN. Their results show high sensitivity and specificity with a diagnosis time of only about 23 s. We employ an improved U-net network to detect rib fractures, and our precision and recall are comparable to those of Zhou et al, but our diagnosis time is significantly shorter, at only about 5 s. Jin et al. [27] used the FracNet algorithm for rib fractures detection and segmentation, achieving a sensitivity of up to 92.9% and 71.5% in Dice for image segmentation, with a diagnosis time of 31 s. FracNet outperforms our model in sensitivity and Dice, but our detection time is only one-sixth of that of FracNet, making it suitable for real-time clinical assistance. Computer-aided diagnosis is a human–computer collaboration approach that improves the performance while reducing the clinical time.

In addition, we tried to adjust the HU value of CT images to obtain 2D images that only kept bones for training and found that this operation damaged the detection effect. It has been proved that the surrounding tissues help identify rib fractures. Unlike natural images, the target in medical images has a closer relationship with surrounding tissues. The addition of the feature information of peripheral tissues will be beneficial for target recognition and segmentation.

There are limitations in our study. Many manual annotations, which are time-consuming and labor-intensive and may be inaccurate, are employed during training. In further studies, we will study how to design an effective self-supervised learning method for the characteristics of medical images. We expect to further improve the accuracy of medical image segmentation and detection by utilizing massive unlabeled images. In conclusion, our detection model can assist clinicians in improving the efficiency of diagnosis in finding rib fractures, which is worth in-depth research.

## Figures and Tables

**Table 1:** Varying θ on the loss function.

θ	0.1	0.15	0.2	0.3	0.4	0.5	0.7	1.0
F1	/	80.01	81.86	79.64	79.98	78.06	78.35	78.15
Recall	/	79.06	81.71	80.61	79.05	80.81	77.99	79.01
Precision	/	80.98	82.02	78.70	80.93	75.49	78.71	77.31

**Table 2:** Performance comparison between the different strategies. “✓” represents that the module has been incorporated into the network for training.

HDDC	CAM	Recall	F1	Precision
		75.56	76.75	77.97
✓		78.41	79.34	80.29
	✓	80.83	79.03	77.31
✓	✓	81.71	81.86	82.02

**Table 3:** Comparison with three networks on the test dataset (5005 2D images).

Model	Recall	Precision	F1	Dice
Unet-34 [43]	75.56	77.97	76.75	49.32
CE-Net [23]	81.66	76.04	78.75	52.03
Unet++ [25]	78.59	81.59	80.06	52.89
RAUNet [24]	80.21	79.06	79.63	51.87
Ours	81.71	82.02	81.86	53.28

## Data Availability

The data presented are available in https://ribfrac.grand-challenge.org/dataset/, (accessed on 25 August 2021).

## Abbreviations

The following abbreviations are used in this manuscript:

CT	Computed tomography
CAM	Combined attention module
HDDC	Hybrid dense dilated convolution module
TP	True positive
FP	False positive
FN	False negative
